# Application of the Electronic Nose Technique to Differentiation between Model Mixtures with COPD Markers

**DOI:** 10.3390/s130405008

**Published:** 2013-04-15

**Authors:** Tomasz Dymerski, Jacek Gębicki, Paulina Wiśniewska, Magdalena Śliwińska, Waldemar Wardencki, Jacek Namieśnik

**Affiliations:** 1 Department of Analytical Chemistry, Chemical Faculty, Gdansk University of Technology, 11/12 G. Narutowicza Str., 80-233 Gdańsk, Poland; E-Mails: tomasz.dymerski@gmail.com (T.D.); p.m.wisniewska@gmail.com (P.W.); m.e.sliwinska@gmail.com (M.S.); waldemar.wardencki@pg.gda.pl (W.W.); jacek.namiesnik@pg.gda.pl (J.N.); 2 Department of Chemical and Process Engineering, Chemical Faculty, Gdansk University of Technology, 11/12 G. Narutowicza Str., 80-233 Gdańsk, Poland

**Keywords:** electronic nose, VOC, MOS, PCA, COPD

## Abstract

The paper presents the potential of an electronic nose technique in the field of fast diagnostics of patients suspected of Chronic Obstructive Pulmonary Disease (COPD). The investigations were performed using a simple electronic nose prototype equipped with a set of six semiconductor sensors manufactured by FIGARO Co. They were aimed at verification of a possibility of differentiation between model reference mixtures with potential COPD markers (N,N-dimethylformamide and N,N-dimethylacetamide). These mixtures contained volatile organic compounds (VOCs) such as acetone, isoprene, carbon disulphide, propan-2-ol, formamide, benzene, toluene, acetonitrile, acetic acid, dimethyl ether, dimethyl sulphide, acrolein, furan, propanol and pyridine, recognized as the components of exhaled air. The model reference mixtures were prepared at three concentration levels—10 ppb, 25 ppb, 50 ppb v/v—of each component, except for the COPD markers. Concentration of the COPD markers in the mixtures was from 0 ppb to 100 ppb v/v. Interpretation of the obtained data employed principal component analysis (PCA). The investigations revealed the usefulness of the electronic device only in the case when the concentration of the COPD markers was twice as high as the concentration of the remaining components of the mixture and for a limited number of basic mixture components.

## 1. Introduction

Each pathological change modifies the chemical composition of the human body, including biological fluids. This fact is utilized in medicine in the diagnosis of various diseases [1]. Chemical analysis of the biological fluids such as blood, urine, saliva, sweat, and exhaled air may allow early recognition of many diseases such as lung cancer, breast cancer, prostate cancer, asthma, tuberculosis, bacterial and viral infections or it may help to monitor blood dialysis processes [2–19]. Unfortunately, the performance of currently applied medical apparatus is limited. They do not allow identification and determination of the concentrations of all the chemical compounds present in the human body, which are critical from a pathological effect standpoint [20]. On the other hand, recently there have been many literature reports on the applications of electronic noses in biomedicine [1–19,21–29]. The electronic nose is a device equipped with a set of non-selective chemical sensors, the response signal of which is correlated with the holistic information obtained from a smell profile of a gas sample or headspace phase of a liquid sample [30–36]. The response signal is a so called fingerprint, which is characteristic for a particular sample (provided the analysis was carried out under precisely defined conditions). Application of the electronic nose allows differentiation between ill and healthy patients based on holistic analysis of the volatile fraction of different biological samples, including exhaled air. Recently medicine has witnessed an increasing interest in cheap, non-invasive and simple diagnostic tests [1,4–13,15–18,20–22,24,25,27,28,37], and for this reason many researchers look for solutions utilizing new diagnostic tools based exclusively on human breath analysis. Volatile organic compounds (VOCs) present in exhaled air may contain information on the internal biochemistry of the human body, which may be useful in terms of identification and description of many diseases. The literature provides information [14,38–40] on applications of the electronic nose technique to diagnosis of chronic obstructive pulmonary disease (COPD), which employed a commercial model Cyranose 320 nose. These investigations confirmed that due to its advantages the technique could be useful and effective as far as breath analysis and differentiation between ill and healthy patients are concerned. COPD is a syndrome revealed as a progressive restriction of air flow through the respiratory system due to malfunction of the lower respiratory system and destruction of the lung parenchyma. The most frequent cause of COPD is exposure to tobacco smoke and to all types of irritant chemical substances during a lifetime [14]. COPD is an incurable disease and treatment is only symptomatic. All treatment activities are aimed at slowing the progression of the disease. This disease can divided into four stages: in the first stage a chronic cough is the predominant symptom, while the second stage is characterized by dyspnoea on exercise. The third stage is deemed a serious one, with escalated dyspnoea and decreased breathing endurance, and finally, in the fourth stage dyspnoea at rest and respiratory distress occur. The main causes of death of patients suffering from COPD are malfunction of the cardiovascular system, lung cancer and respiratory distress. The aforementioned facts illustrate how important early identification of COPD is, so patients' life comfort can be improved as well as disease progress inhibited (disease retardation). Statistical data show the scale of the problem. For instance, in the United States of America COPD is the fourth on the list of death causes due to diseases.

This paper presents model studies on mixtures of reference substances based on the volatile organic compounds that are components of human breath. Some of these compounds, namely N,N-dimethyl-formamide and N,N-dimethylacetamide, were mentioned in the literature as potential COPD markers [5,41,42]. A 6-sensor electronic nose prototype was utilized in the investigations. The set of sensors comprised cheap semiconductor sensors manufactured by Figaro Co. The aim of the studies was verification of the usefulness of the prototype in differentiation between mixtures of reference substances containing the COPD markers at different concentration levels and mixtures without these substances. How complexity of the matrix influences the differentiation ability was also checked. An intention of the authors was to find an answer to the question: can this type of device (cheap and simple—not equipped with higher sensitivity (and cost) SAW/BAW type sensors) be applied in practice? Moreover, performed investigations could be an impulse for future development and wide implementation of cheap, fast and non-invasive electronic nose techniques in the field of COPD identification.

## 2. Experimental Section

### 2.1. Measurement Set-Up

**Figure 1.**
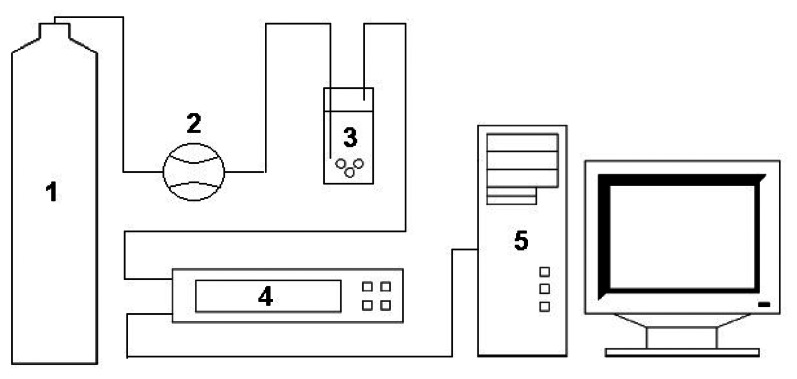
Experimental set-up for analysis of volatile fraction of reference gaseous mixtures consisting in: 1—bottle with carrier gas, 2—flow meter, 3—scrubber, 4—prototype of electronic nose, 5—PC.

Figure 1 presents a scheme of the measurement set-up consisting of a container with carrier gas, a flow meter by Tecfluid, a “petit coat” scrubber, a prototype of electronic nose and a PC computer. The carrier gas was compressed air of N5.0 purity (Linde Gaz Poland Ltd.) All components of the measurement set-up, from the gas container to the electronic nose device were connected via a Teflon tube of diameter Φ 4 mm.

### 2.2. Structure of a Prototype of Electronic Nose

**Figure 2.**
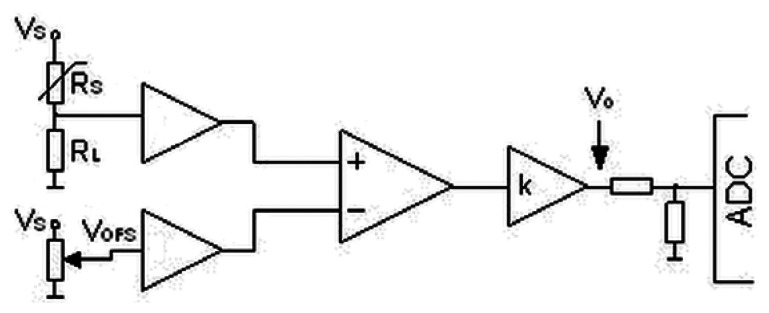
Scheme of integrated circuit.

The prototype of the electronic nose was built from six commercial, semiconductor sensors (TGS 880, TGS 825, TGS 826, TGS 822, TGS 2610, TGS 2602 by Figaro Co.). All internal parts of the prototype: scrubber, connecting tubes and module with the sensors were in a thermostatic casing in order to provide stable measurement conditions. The temperature was maintained at 36.6 ± 0.3 °C. Relative humidity of air inside the module with the sensors was 90 ± 1%. A conversion of the sensors' output signals to digital signals was accomplished via a dedicated miniaturized integrated circuit. This circuit (Figure 2) consisted of a sensor of resistance R_s_ (operating within a voltage divider V_s_ = 5 V), termination resistance selected for each sensor R_L_, amplifying course with adjustable amplification k and zero system with adjustable voltage offset V_OFS_. The aim of the circuit was to convert changes of sensor resistance into voltage signal measurable by an analogue-to-digital converter (ADC).

The resultant voltage signal V_o_ can be described by the Equation (1):

(1) $$V_{o}=k\;\left(V_{s}\;\frac{R_{L}}{R_{L}+R_{s}}-V_{\text{OFS}}\right)$$

and its changes in the complete measurement range of the converter correspond to the complete range of changes of sensor resistance. Obtained voltage was digitally converted into a scale from 0 to 14 bits. During interpretation of the results a function S/S_max_ of the sensor signal was utilized, which is a ratio of the voltage from a particular sensor to the maximum signal. This is digital information (voltage acquired from a particular sensor) divided by 14 bits. Measurement data were collected and recorded at 1 s intervals starting from the measurement onset and subjected to preliminary processing using dedicated software. Reproducibility of 98% of the results obtained using the e-nose prototype was within the range of 3.8–7.4% Coefficient of Variation (CV). In order to evaluate the coefficients of variation five analyses were performed for each mixture. The structural elements of the e-nose device, the modules for thermal stabilization of sample during barbotage, the modules for temperature and relative humidity stabilization of air containing analyte are the subject of a patent application. Interpretation of the results of performed analysis was carried out with commercially available software SAS Enterprise 4.3 with an implemented algorithm for chemometric calculations utilizing the principal component analysis (PCA) approach.

### 2.3. Reagents

Fifteen reference substances from the VOCs group: acetone, isoprene, carbon disulphide, propan-2-ol, formamide, benzene, toluene, acetonitrile, acetic acid, dimethyl ether, dimethyl sulphide, acrolein, furan, propanol and pyridine, being the main components of human breath [20,41], were utilized in the studies and defined in this paper as the basic components of the reference mixtures. Moreover, two substances: N,N-dimethylformamide and N,N-dimethylacetamide were used as the COPD markers [41]. The aforementioned compounds were of p.f.a. grade (Sigma-Aldrich). The solvent was deionised water from a Mili-Q A 10 device by Millipore Co.

### 2.4. Sample Preparation to Analysis

Nine different reference mixtures were prepared for investigation and marked: A 5, A 10, A 15, B 5, B 10, B 15, C 5, C 10, C 15 (Table 1).

**Figure 3.**
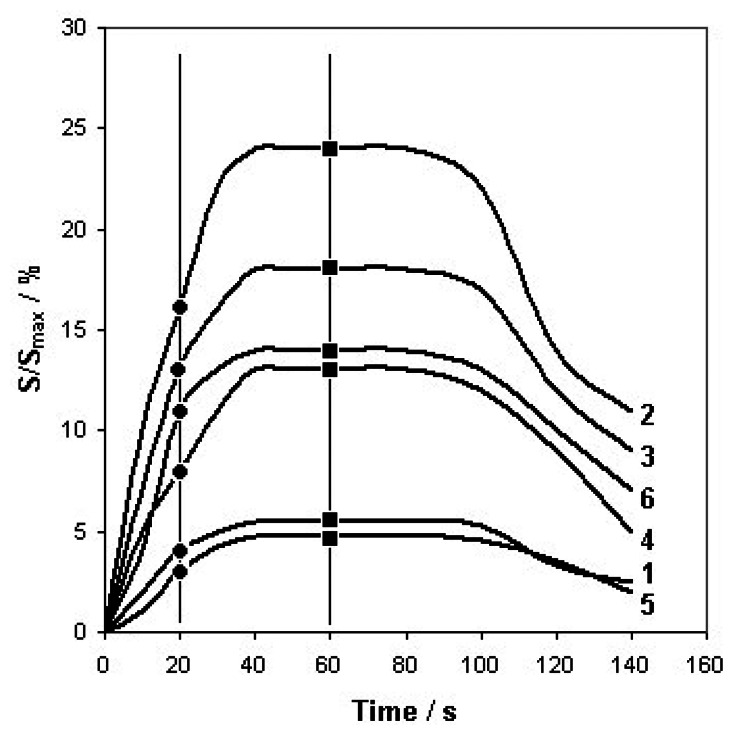
Dependence between sensor signals and barbotage time for A 5 type mixture. Time instants, at which PCA was performed: 20 s (●), 60 s (■). Sensors: 1–TGS 880, 2–TGS 825, 3–TGS 826, 4–TGS 822, 5–TGS 2610, 6–TGS 2602.

**Figure 4.**
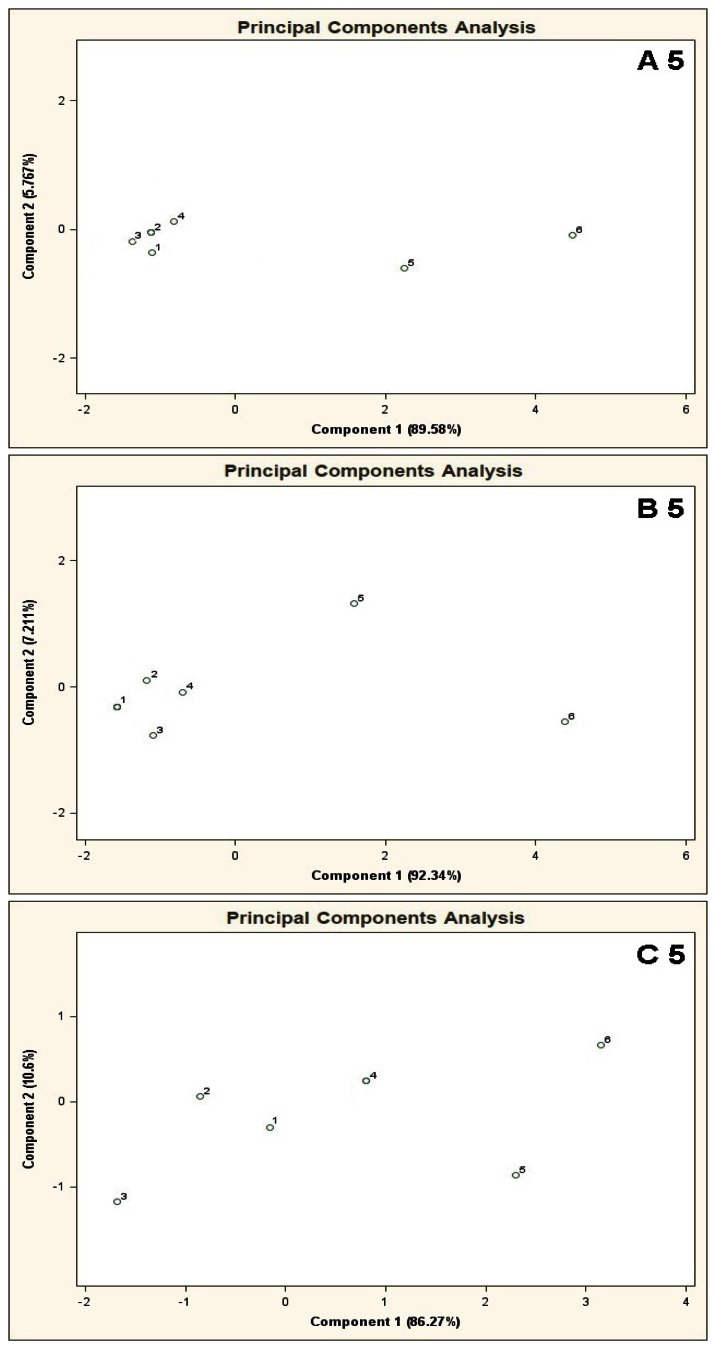
Result of PCA for the mixtures of type **A 5**, **B 5**, **C 5**, where A 5, B 5, C 5 are the aqueous solutions of the basic components (acetone, isoprene, carbon disulphide, propan-2-ol, formamide) having the concentration of each component in the mixture equal 10 ppb, 25 ppb and 50 ppb v/v, respectively. 1–6 are the mixtures of reference substances with the COPD markers of the respective concentrations: 0 ppb, 5 ppb, 10 ppb, 25 ppb, 50 ppb, 100 ppb v/v. Measurement was obtained for time instant 60 s.

**Figure 5.**
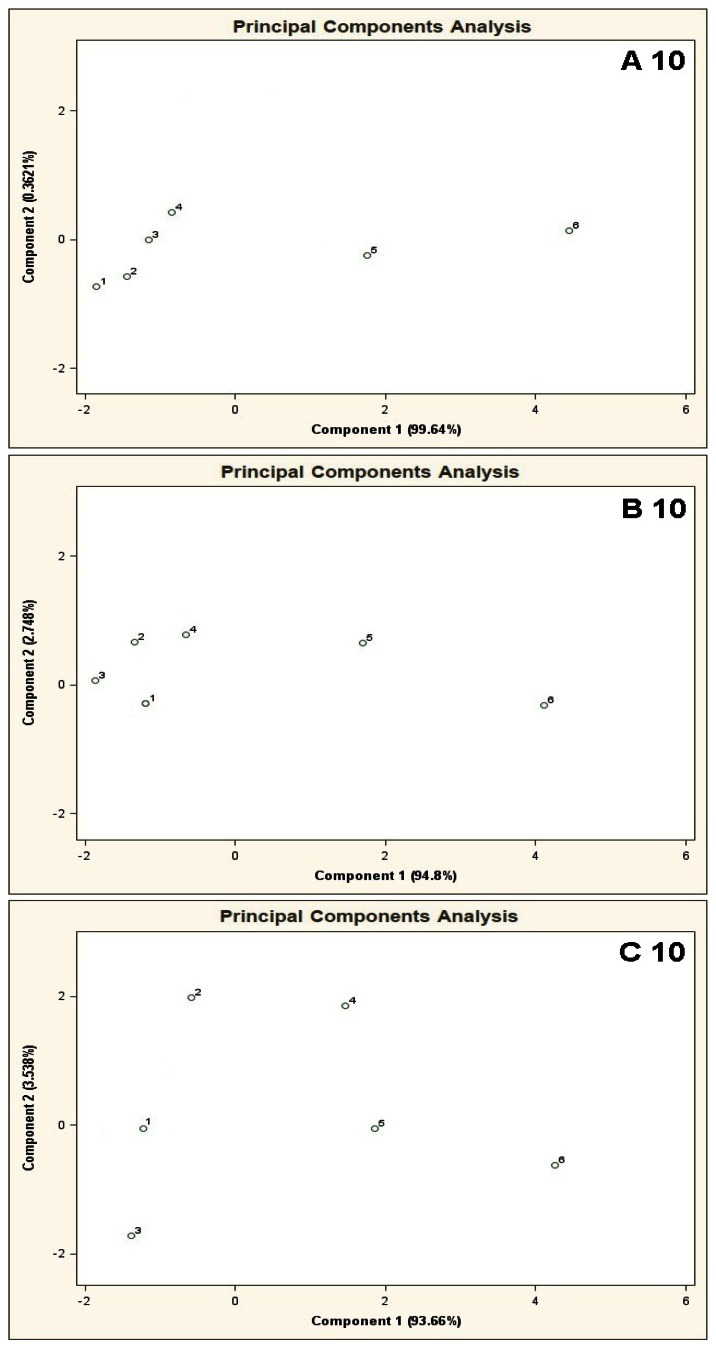
Result of PCA for the mixtures of type **A 10**, **B 10**, **C 10**, where A 10, B 10, C 10 are the aqueous solutions of the basic components (acetone, isoprene, carbon disulphide, propan-2-ol, formamide, benzene, toluene, acetonitrile, acetic acid, dimethyl ether) having the concentration of each component in the mixture equal 10 ppb, 25 ppb and 50 ppb v/v, respectively. 1–6 are the mixtures of reference substances with the COPD markers of the respective concentrations: 0 ppb, 5 ppb, 10 ppb, 25 ppb, 50 ppb, 100 ppb v/v. Measurement was obtained for time instant 60 s.

**Figure 6.**
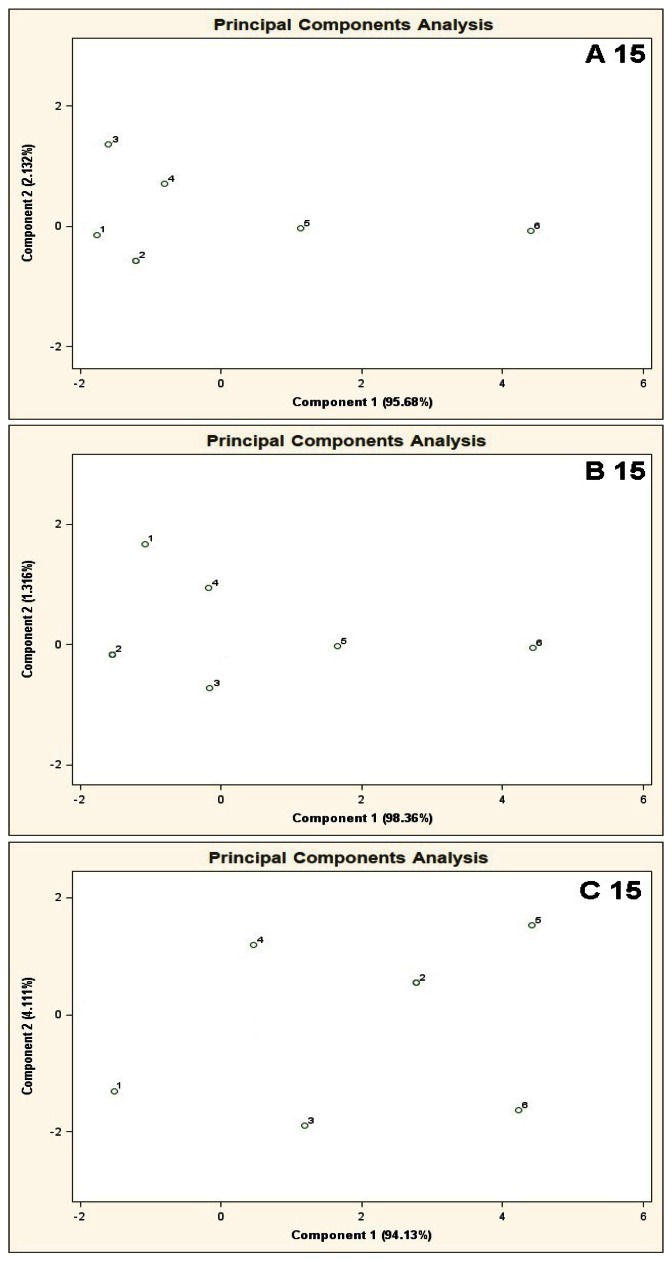
Result of PCA for the mixtures of type **A 15**, **B 15**, **C 15**, where A 15, B 15, C 15 are the aqueous solutions of the basic components (acetone, isoprene, carbon disulphide, propan-2-ol, formamide, benzene, toluene, acetonitrile, acetic acid, dimethyl ether, dimethyl sulphide, acrolein, furane, propanol, pyridine) having the concentration of each component in the mixture equal 10 ppb, 25 ppb and 50 ppb v/v, respectively. 1–6 are the mixtures of reference substances with the COPD markers of the respective concentrations: 0 ppb, 5 ppb, 10 ppb, 25 ppb, 50 ppb, 100 ppb v/v. Measurement was obtained for time instant 60 s.

**Figure 7.**
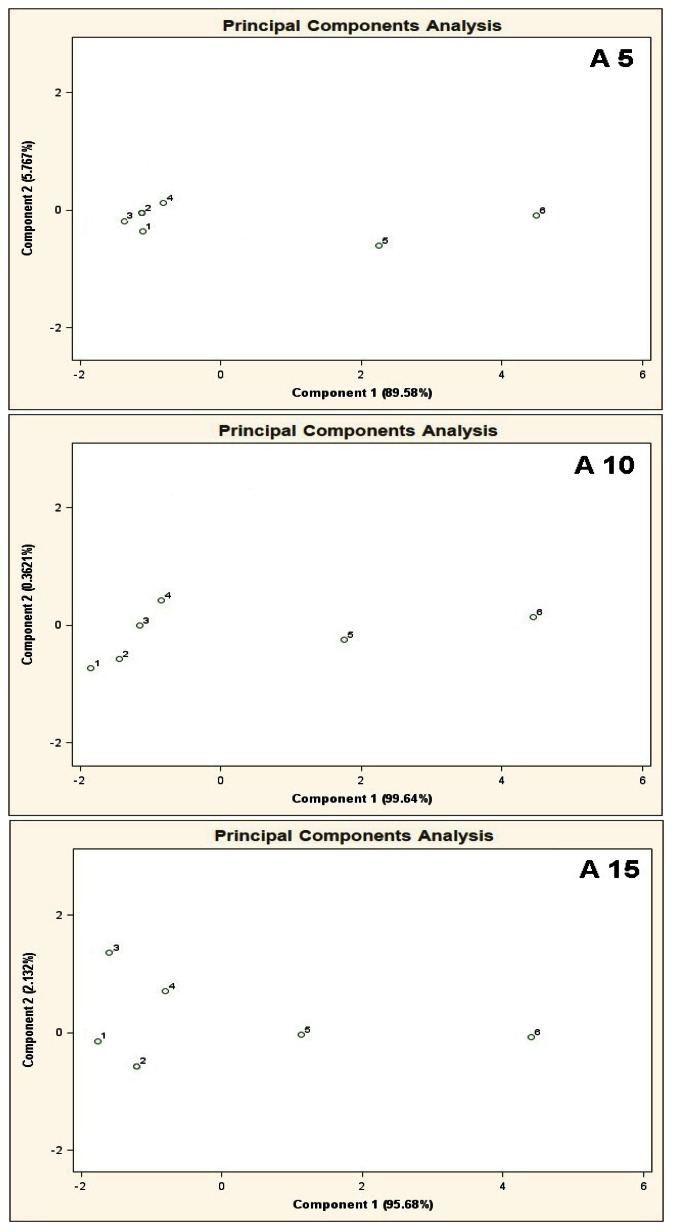
Result of PCA for the mixtures of type **A 5**, **A 10**, **A 15**, where A 5, A 10, A 15 are the aqueous solutions of the basic components of amount 5, 10 and 15 in the mixtures of concentration 10 ppb v/v for each component. 1–6 are the mixtures of reference substances with the COPD markers of the respective concentrations: 0 ppb, 5 ppb, 10 ppb, 25 ppb, 50 ppb, 100 ppb v/v. Measurement was obtained for time instant 60 s.

**Figure 8.**
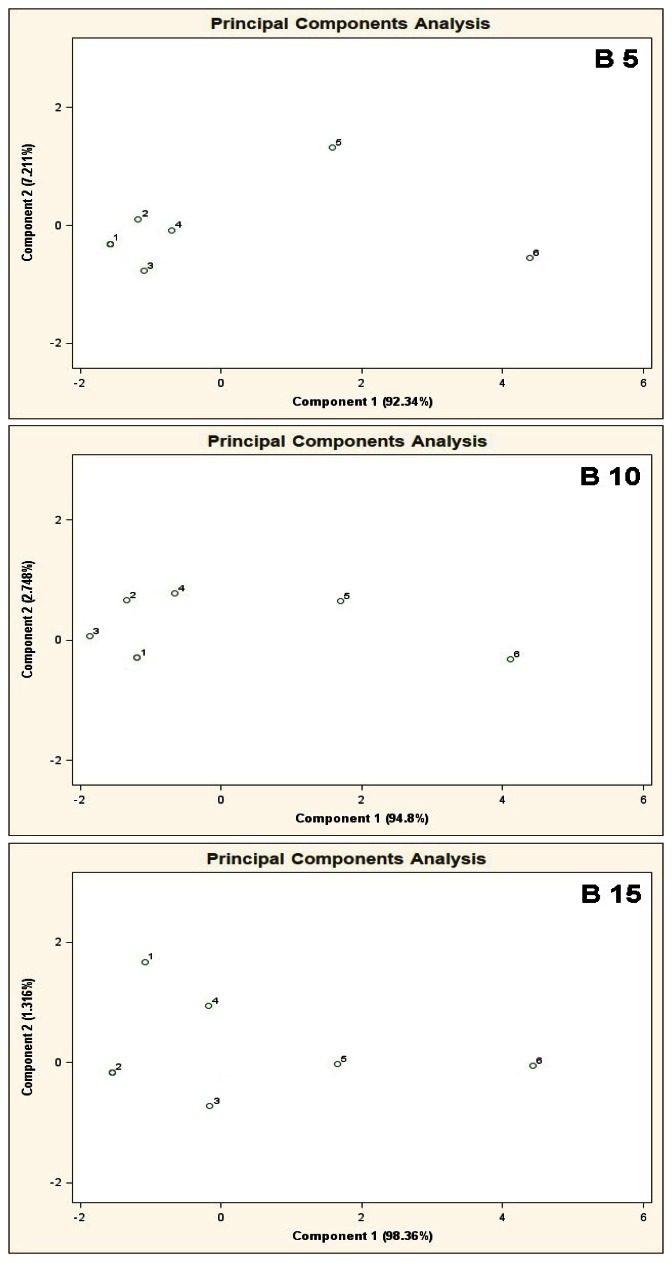
Result of PCA for the mixtures of type **B 5**, **B 10**, **B 15**, where B 5, B 10, B 15 are the aqueous solutions of the basic components of amount 5, 10 and 15 in the mixtures of concentration 25 ppb v/v for each component. 1–6 are the mixtures of reference substances with the COPD markers of the respective concentrations: 0 ppb, 5 ppb, 10 ppb, 25 ppb, 50 ppb, 100 ppb v/v. Measurement was obtained for time instant 60 s.

Each basic component was present at the following concentration levels: 10 ppb v/v for mixture type A, 25 ppb v/v for mixture type B, 50 ppb v/v for mixture type C. The numbers 5, 10 and 15 in a PCA result name denote the number of basic components in particular mixtures (excluding the COPD markers), where: 5-acetone, isoprene, carbon disulphide, propan-2-ol, formamide, 10-acetone, isoprene, carbon disulphide, propan-2-ol, formamide, benzene, toluene, acetonitrile, acetic acid, dimethyl ether, 15-acetone, isoprene, carbon disulphide, propan-2-ol, formamide, benzene, toluene, acetonitrile, acetic acid, dimethyl ether, dimethyl sulphide, acrolein, furan, propanol, pyridine. Moreover, the concentrations of each COPD marker (N,N-dimethyl-formamide and N,N-dimethylacetamide) in the reference mixtures were: 0, 5, 10, 25, 50, 100 ppb v/v. These mixtures were denoted in PCA results by numbers from 1 to 6. The total number of the mixtures prepared was 54. The investigations were carried out for three months. PCA results presented in Figures 3, 4, 5, 6, 7 and 8 illustrate data averaged over five measurements for each measurement series.

### 2.5. Optimization of Operation Parameters of Electronic Nose Prototype

The barbotage process resulted in a transfer of the analytes from a liquid phase to a gas phase. Thermodynamic equilibrium between the liquid and gas phase established in given conditions (temperature, type of analyte), time of barbotage process and flow rate of the inert gas determine concentration of the analyte in the gas phase. Concentration of a given analyte in the gas phase can be calculated from Equation (2):

(2) $$c_{G}=\frac{c_{L0}}{K}\exp\left(-\frac{Qt}{KV_{L}}\right)$$

where c_L0_—initial analyte concentration in liquid phase, c_G_—instantaneous analyte concentration in gas phase, Q—volumetric flow rate of inert gas, t—time of barbotage, V_L_—volume of liquid phase, K—partition coefficient described by Equation (3):

(3) $$K=\frac{c_{LR}}{c_{GR}}$$

where c_LR_—analyte concentration in liquid phase being in thermodynamic equilibrium with gas phase, c_GR_—analyte concentration in gas phase being in thermodynamic equilibrium with liquid phase. The partition coefficient *K* is a quantitative measure of thermodynamic equilibrium and higher value of this coefficient means lower concentration of the analyte in the gas phase.

The parameters Q and t were optimized in order to obtain maximum and constant sensors response time. Volumetric flow rate was 5 dm^3^/h and time of barbotage (single analysis) was equal 1 min. Additionally, the results of PCA of the sensor signals after 20 s were presented in order to verify the optimization method adopted. Figure 3 presents dependence between the signal of six sensors and time of barbotage (A 5 type mixture) with the time instants, at which the PCA was performed.

## 3. Results and Discussion

Figure 4 presents PCA results for the mixtures of type A 5, B 5, C 5 where the points from 1 to 6 denote the mixtures of reference substances, in which the COPD markers are present at the concentrations of 0, 5, 10, 25, 50, 100 ppb v/v, respectively (this notation applies also to the plots from Figure 5 to Figure 11). It can be noticed that the results of A 5 and B 5 analyses differ from the C 5 analysis result. Low concentration of the basic components in case of A 5 (10 ppb v/v) and B 5 (25 ppb v/v) make it possible to differentiate the mixtures 1–4 from the mixtures 5, 6. Such a situation is not possible in case of the C 5 analysis results. For analyses A 5 and B 5 the distances between the point 1 and the points 2–4 are much smaller as compared to the distances between the point 1 and the points 5,6. The interpretation can be as follows: the device is able to differentiate the samples containing the COPD markers at higher concentration levels (50 and 100 ppb v/v) in the samples, in which the concentration of the markers is comparable to the concentration of the basic components or where the concentration of the markers equals 0 ppb v/v. The C 5 analysis result shows that the distances between the points 1–6 are similar, which means differentiation between the mixtures having comparable concentrations of the basic components and the COPD markers is impossible.

Applying a cluster analysis to description of internal structure of the multidimensional space of features we utilized a direct method (sphere method) employing a distance matrix. A distance between objects *d^T^* in the multidimensional space of features was estimated based on absolute value of tangent of the angle between features' vectors given by Equation (4):

(4) $$d^{T}=\sqrt{\frac{1-r^{2}}{r^{2}}}$$

where *r* is the coefficient of correlation between feature pairs.

The sphere method relies on an assumption that the objects in a cluster focus around certain central point. The first stage of analysis consists in finding the object within the cluster, which is the closest to the central point. In practice, for each object one defines the number of remaining objects present within the distance smaller than R, it means inside the hyper-sphere of radius R. The radius of the hyper-sphere is determined as the biggest *d^T^* of the smallest distances between the objects *i*,*j* = 1,….n following Equation (5):

(5) $$R=\max|\min(d_{ij}^{T})|$$

The results of sphere method analysis contained in Table 2 show that for A 5 type mixture there are three clusters identified as the mixture 1–4 and two separated objects: mixtures 5 and 6. In the case of B 5 type mixture there are two clusters: mixture 1–4 and mixtures 5 and 6. Analysis of C 5 type mixture suggests the presence of one cluster only for all the mixtures 1–6.

The results of cluster analysis performed with the sphere method are convergent with the observation of the PCA planes and distribution of the points 1–6 over these planes. Figure 5 illustrates PCA results for the mixtures of type A 10, B 10, C 10. In this case one can also notice that the results of A 10 and B 10 analyses differ from the C 10 analysis result. The points 1–4 for A 10 and B 10 analyses are clustered around each other and very distant from the points 5 and 6. It means that when the concentration of the COPD markers is higher than the concentration of the basic components differentiation between the mixtures is possible. No influence of the number of basic components on analysis results was observed (there was similarity between the A 5-A 10 results, as well as between B 5 and B 10). In the case of C 10 analysis differentiation of the mixtures with various concentrations of the COPD markers was not possible. Point 5 lies within similar distance from the point 1 (the mixture without the COPD markers) as the points 2, 3, 4. It makes differentiation between the mixture 5 and the mixtures 1–4 impossible. A ratio of the distance between the point 1 and the points 2–5 to the distance between the point 1 and the point 6 is close to unity. It means high uncertainty of differentiation between the mixture 6 and the remaining ones. The cluster analysis (results in Table 2) confirms presence of two clusters for the mixtures of type A 10 and B 10, which contain the objects representing the mixtures 1–4 and the mixtures 5 and 6. In the case of C 10 type mixture the calculations reveal presence of one cluster only corresponding to the entire mixture 1–6.

Figure 6 shows PCA results for the mixtures of type A 15, B 15, C 15. The result of A 15 analysis makes it possible to identify mixture 6 only. The distance between the point 6 and the point 1 is much bigger (more than two times) the distances between the point 1 and the remaining points. In the case of B 15 analysis differentiation between the mixture 6 and the remaining ones is burdened with relatively high uncertainty. The distance from the point 1 to the point 6 is ca. one and a half times bigger than the distance between the points 1 and 5 (the point 5 is in a vicinity of the points 1–4 and is the closest to the point 6). The cluster analysis does not confirm the presence of two clusters for A 15 type mixture, however it is convergent with the information obtained via PCA of the mixtures of type B 15 and C 15 where existence of only one cluster was discovered, which made it impossible to differentiate between the mixtures 1-6 (information contained in Table 2).

PCA results for the mixtures of type A 5, A 10, A 15 are presented in Figure 7. Bigger number of the basic components, keeping their concentration 10 ppb v/v constant, deteriorated differentiation of the mixtures 5 and 6 from the remaining ones. The result was higher scatter of the points 1–4 on a two-dimensional PCA plane and a decrease in the distance between these points and the points 5, 6.

PCA results for the mixtures of type B 5, B 10, B 15 are illustrated in Figure 8. The results of these analyses are characterized by close similarity to the results presented in Figure 7, where increased number of the basic components deteriorates differentiation of the mixtures 5, 6 from the mixtures 1–4.

**Figure 9.**
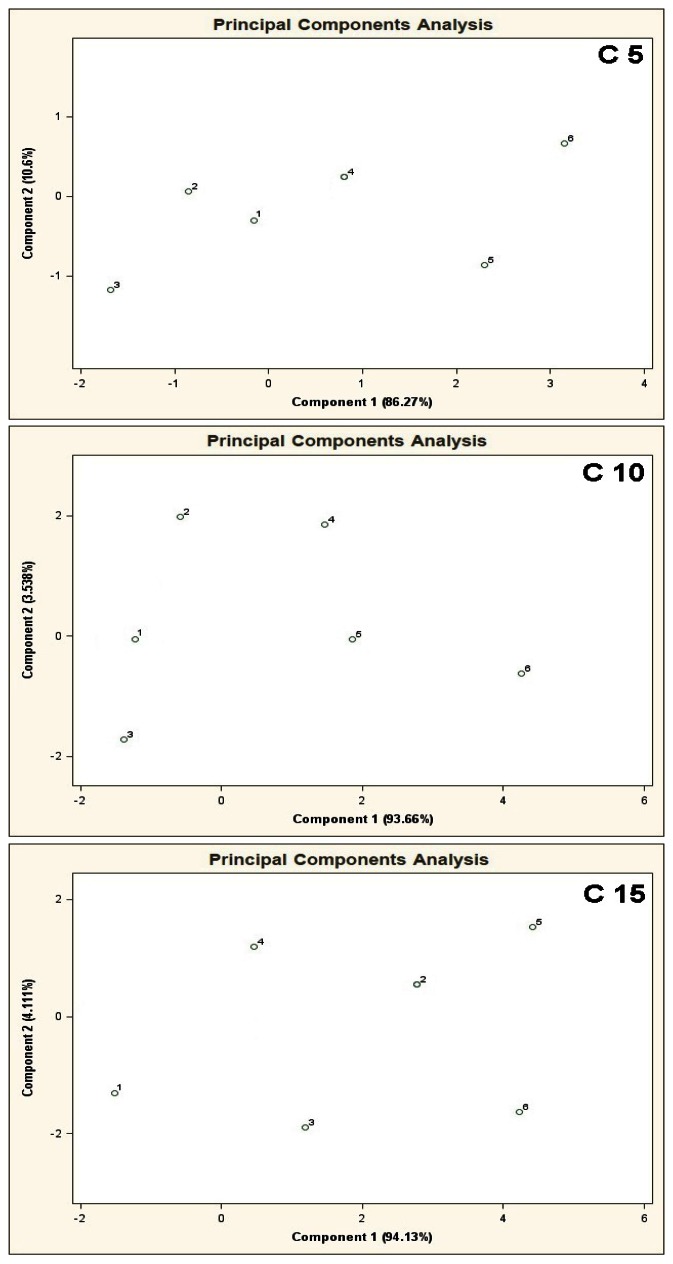
Result of PCA for the mixtures of type **C 5**, **C 10**, **C 15**, where C 5, C 10, C 15 are the aqueous solutions of the basic components of amount 5, 10 and 15 in the mixtures of concentration 50 ppb v/v for each component. 1–6 are the mixtures of reference substances with the COPD markers of the respective concentrations: 0 ppb, 5 ppb, 10 ppb, 25 ppb, 50 ppb, 100 ppb v/v. Measurement was obtained for time instant 60 s.

PCA results for the mixtures of type C 5, C 10, C 15 are shown in Figure 9. In case of high concentration of the basic components (50 ppb v/v) with respect to the COPD markers in the mixtures 1–6 (from 0 ppb to 100 ppb v/v) it is not possible to differentiate between the mixtures 1–6.

**Figure 10.**
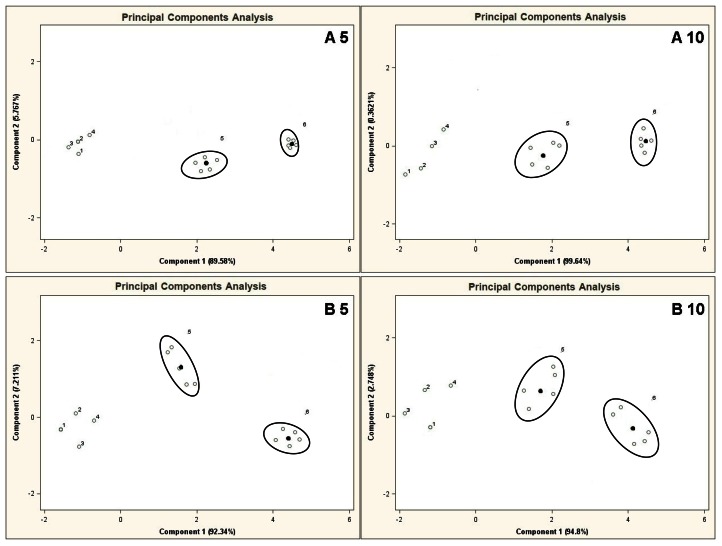
Result of PCA for the mixtures of type **A 5**, **B 5**, **A 10**, **B 10** where the points for the mixtures 5 and 6 represent mean values surrounded by a single result. Measurement was obtained for time instant 60 s.

Figure 10 illustrates the PCA results for the mixtures A 5, B 5, A 10, B 10, presenting all results of analyses for the mixtures 5 and 6. Black points 5 and 6 marked on the PC planes are the averaged results from five measurements. It can be seen that the region of PC plane attributed to the mixture 5 is distinguishable from the region where the points representing the mixture 6 are. Such a separation excludes relatively any high correlation between them (presence of information redundancy), which in turn is observed for the remaining points 1–4 (lack of clear separation of points).

**Figure 11.**
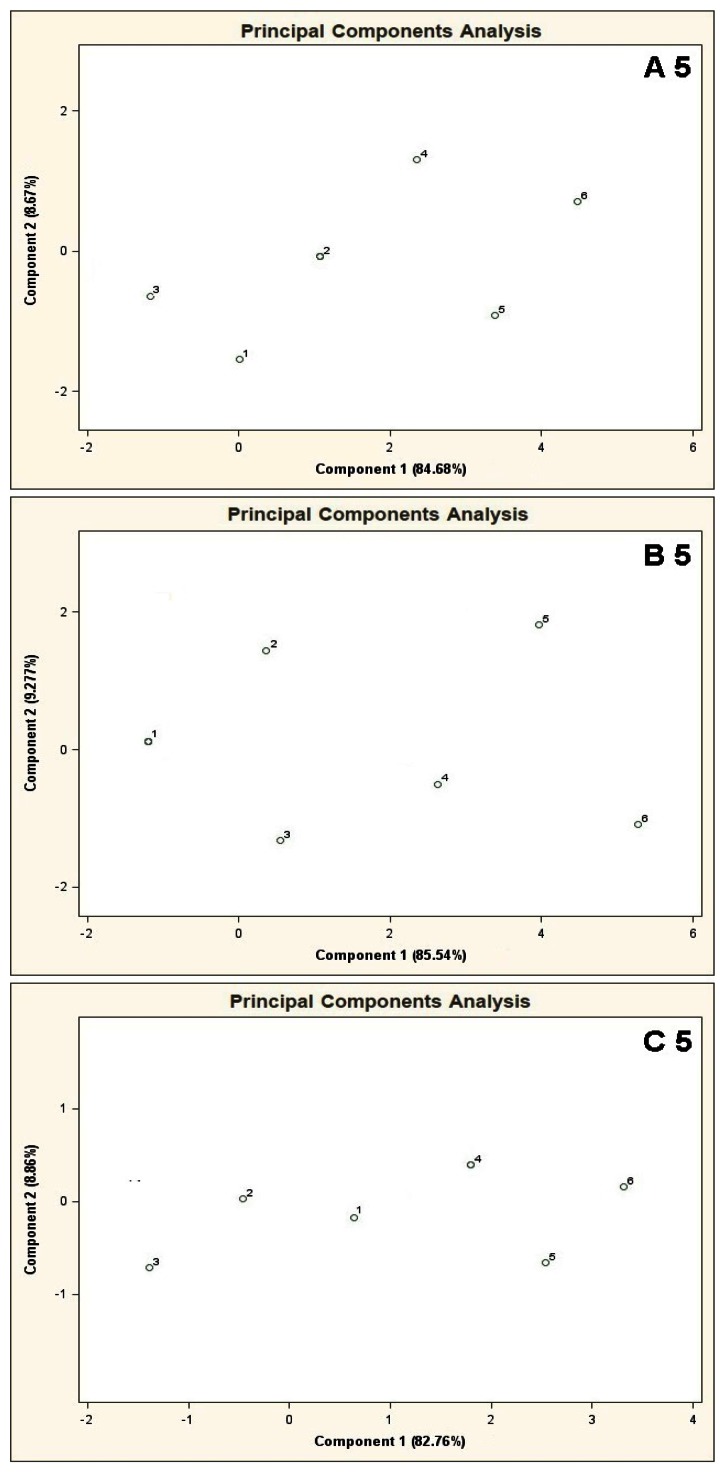
Result of PCA for the mixtures of type **A 5**, **B 5**, **C 5**. Measurement was obtained for time instant 20 s.

Figure 11 illustrates the PCA results (for time instant 20 s) for the mixtures of type A 5, B 5, C 5. This time no separate points 5 and 6 were observed on the PC plane, unlike for the mixtures of type A 5 and B 5 presented in Figure 4. The reason could be a relative low content or lack of the potential COPD markers in the gas mixture prepared. Relatively low volatility of the potential COPD markers as compared to the other components of the mixture and too short time of barbotage could be the reason of a delay in appearance of noticeable changes in the sensor signal and thus the lack of characteristic separated points in the PC plane.

## 4. Conclusions

The aim of the investigations was verification of the potential usefulness of a cheap and fast-operating electronic nose prototype in the differentiation of reference mixtures containing COPD markers. The device was equipped with a set of six semiconductor sensors manufactured by Figaro Co. Gaseous samples were prepared by the barbotage process from aqueous solutions containing the compounds belonging to the VOCs group. The time of a single analysis of prepared liquid sample was 1 min and the total time of a single analysis with chemometric data interpretation amounted to 2.5 min.

**Figure 12.**
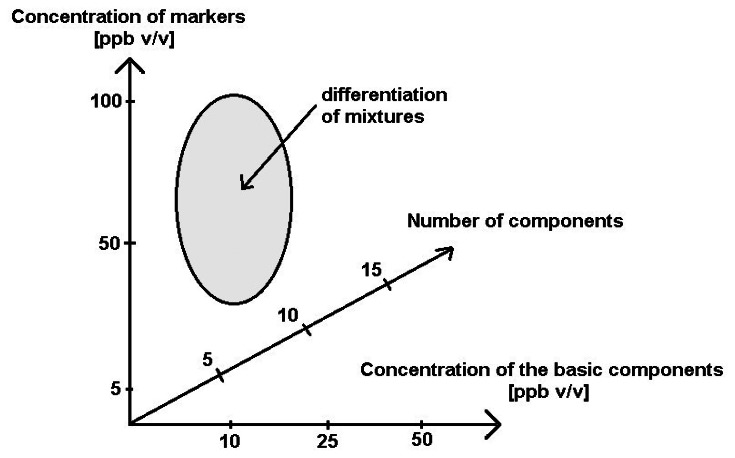
Schematic presentation of differentiation between the mixtures with COPD markers using electronic nose device.

The investigations revealed a possibility of differentiation between the mixtures with the COPD markers in the case when a number of the basic components was 5 and 10, when their concentration was at the level 10 ppb and 25 ppb v/v, and when the concentration of the markers in these mixtures was at the level 50 ppb and 100 ppb v/v. It was confirmed by the cluster analysis performed using the sphere method. Higher concentrations of the basic components in the mixtures and increased number of these components made it impossible to differentiate between the mixtures 1–6. The electronic nose device proved its usefulness only when the concentration of the COPD markers was at least two times higher than the concentration of the mixtures and only for limited number of the basic components. These conclusions are illustrated in Figure 12.

Human breath contains more than 200 compounds from the VOCs group [43]. The majority of them are present at trace concentrations, whereas there are from a few to several main components of exhaled air (*i.e.*, acetone, isoprene, carbon disulphide, propan-2-ol). The authors observed than it is the concentration of particular components of the mixture and not their number that had a bigger influence on the differentiation ability. This fact suggests that at this stage of research the device could be applied in case of the patient suffering from advanced stages of COPD. Early identification of COPD using the electronic nose device requires application of SAW/BAW type sensors [8] or sensor matrixes with carbon nanotubes like those presented by Haick *et al* [44,45], which are characterized by higher sensitivity and selectivity towards amide compounds.

## Figures and Tables

**Table 1.** Composition of reference mixtures.

**Designation of the Mixtures**	**Number of Components**	**Concentration of Each Component**	**Components**
A 5	5	10 ppb v/v	acetone, isoprene, carbon disulphide, propan-2-ol, formamide
B 5	5	25 ppb v/v	acetone, isoprene, carbon disulphide, propan-2-ol, formamide
C 5	5	50 ppb v/v	acetone, isoprene, carbon disulphide, propan-2-ol, formamide
A 10	10	10 ppb v/v	acetone, isoprene, carbon disulphide, propan-2-ol, formamide, benzene, toluene, acetonitrile, acetic acid, dimethyl ether
B 10	10	25 ppb v/v	acetone, isoprene, carbon disulphide, propan-2-ol, formamide, benzene, toluene, acetonitrile, acetic acid, dimethyl ether
C 10	10	50 ppb v/v	acetone, isoprene, carbon disulphide, propan-2-ol, formamide, benzene, toluene, acetonitrile, acetic acid, dimethyl ether
A 15	15	10 ppb v/v	acetone, isoprene, carbon disulphide, propan-2-ol, formamide, benzene, toluene, acetonitrile, acetic acid, dimethyl ether, dimethyl sulphide, acrolein, furan, propanol, pyridine
B 15	15	25 ppb v/v	acetone, isoprene, carbon disulphide, propan-2-ol, formamide, benzene, toluene, acetonitrile, acetic acid, dimethyl ether, dimethyl sulphide, acrolein, furan, propanol, pyridine
C 15	15	50 ppb v/v	acetone, isoprene, carbon disulphide, propan-2-ol, formamide, benzene, toluene, acetonitrile, acetic acid, dimethyl ether, dimethyl sulphide, acrolein, furan, propanol, pyridine

**Table 2.** Summary information obtained based on cluster analysis employing sphere method to evaluation of the number of object clusters in multidimensional space of features.

**Mixture**	**Number of Balls (clusters)**	**Content Ball 1**	**Content Ball 2**	**Content Ball 3**
A 5	3	mixture 1–4	mixture 5	mixture 6
B 5	2	mixture 1–4	Mixtures (5,6)	lack
C 5	1	mixture 1–6	lack	lack
A 10	2	mixture 1–4	mixtures (5,6)	lack
B 10	2	mixture 1–4	mixtures (5,6)	lack
C 10	1	mixture 1–6	lack	lack
A 15	1	mixture 1–6	lack	lack
B 15	1	mixture 1–6	lack	lack
C 15	1	mixture 1–6	lack	lack
